# Computer and telephone delivered interventions to support caregivers of people with dementia: a systematic review of research output and quality

**DOI:** 10.1186/s12877-017-0654-6

**Published:** 2017-11-16

**Authors:** Amy Waller, Sophie Dilworth, Elise Mansfield, Rob Sanson-Fisher

**Affiliations:** 1 Health Behaviour Research Collaborative, School of Medicine and Public Health, University of Newcastle, Callaghan, NSW 2308 Australia; 2 Hunter Medical Research Institute, Newcastle, NSW 2305 Australia; 3 Priority Research Centre for Health Behaviour, University of Newcastle, Callaghan, NSW 2308 Australia

**Keywords:** Dementia, eHealth, Telephone, Caregiver, Technology, Internet

## Abstract

**Background:**

To assess the scope, volume and quality of research on the acceptability, utilisation and effectiveness of telephone- and computer-delivered interventions for caregivers of people living with dementia.

**Methods:**

Medline, EMBASE, CINAHL and Cochrane databases were searched (Jan 1990 – Dec 2016). Eligible papers were classified as data-based descriptive, measurement or intervention studies. Intervention studies were first categorised according to mode of delivery (e.g. telephone, computer); then assessed against the Effective Practice and Organisation of Care (EPOC) methodological criteria for research design. Impact on health-related outcomes; and the acceptability, feasibility and utilisation of interventions were also assessed.

**Results:**

The number of publications increased by 13% each year (*p* < 0.001). Half were descriptive studies (*n* = 92, 50%) describing caregiver views on acceptability, access or utilization of technology. The remainder (*n* = 89, 48%) reported on interventions designed to improve caregiver outcomes. Only 34 met EPOC design criteria. Interventions were delivered via computer (*n* = 10), multiple modalities (*n* = 9) or telephone (*n* = 15). Interventions that incorporated various elements of psycho-education, peer support, skills training and health assessments led to improvements in caregiver wellbeing. While largely acceptable, utilisation of computer-based interventions was variable, with use often decreasing over time.

**Conclusion:**

Interventions delivered via telephone and computer have the potential to augment existing dementia care. High-quality trials are required to make clear recommendations about the types of interventions that are most effective. Those that provide caregivers with: access to practical strategies to manage care of the person with dementia and their own wellbeing, advice and support from peers and/or clinicians; and that target the dyad should be explored.

**Electronic supplementary material:**

The online version of this article (10.1186/s12877-017-0654-6) contains supplementary material, which is available to authorized users.

## Background

Dementia can have complex health, practical and social consequences for the person living with dementia and their family. Most people with dementia (70%) live in the community [1]. People with dementia may report a range of physical, psychosocial and practical concerns. If left unmet, these concerns may adversely impact quality of life [2, 3]. Informal caregivers are often called on to help manage activities of daily living, behavioural and psychological symptoms; and organise care and provide emotional support [4]. Caregiving has been linked to health and psychosocial concerns [5].

The need to improve care of people with dementia and to better support their caregivers across multiple domains has been emphasised [6]. These include the need for improvements in the quality of information available to this population; the implementation of care plans tailored to individual needs; and greater support for caregivers to ensure they are able to respond to the concerns of the person with dementia while managing their own health [6]. Limited awareness within the community about services available to provide support; insufficient provider time, skills or resources to deliver care [7, 8]; and inconsistencies in monitoring and management of physical, psychosocial and practical concerns can result in fragmented dementia care [6].

Health resources are stretched to capacity, and there are a range of geographical, practical and system barriers to optimal dementia care [9]. The subsequent demands placed on caregivers of people with dementia means face-to-face support services can be burdensome or impractical. Hence, the delivery of services via alternative means, such as by computer or telephone, may allow individualised support of a broad cross-section of consumers in a timely way. More than half of people aged over 65 [10–12]; and up to 80% of caregivers of older adults are internet users [13]. These modes of delivery can facilitate the provision of credible information and resources that enable consumers to exercise control over their lives, and promote efficient communication between consumers and health care teams. Systematic reviews highlight the potential benefits of interventions delivered by professionals to caregivers of people with dementia [14]. Others describe benefits of interventions delivered via telephone or computer for other patient groups and their caregivers, including improvements to quality of life, perceived support, knowledge and satisfaction (e.g. [15–17]). The need for further research is emphasised, particularly given the rapidly changing technology field. Determining the effectiveness of interventions for specific caregiver groups is important, as there may be variation in complexity and severity of needs experienced by caregivers and the people they support. While there has been some synthesis of the evidence for computer and telephone interventions designed for caregivers of people with dementia [18, 19], none have examined the type, quality and impact of research.

To provide a comprehensive overview, several aspects of the available literature should be explored. The volume and type of research represents an indication of broad research effort. These indices can identify gaps that can be targeted by further research to guide practice and policy. Establishing the quality of the research [20] can reveal areas where increased capacity through further training or allocation of resources may be required. The aims of this review were to examine the extent to which computer and telephone delivered interventions for caregivers of people with dementia have been examined in the literature, including the: 1) volume and type of data-based publications; 2) methodological quality of intervention studies according to Effective Practice and Organisation of Care (EPOC) criteria; and 3) the effectiveness, acceptability and utilisation of interventions in studies that met minimum criteria for quality.

## Methods

### Definition of interventions

Included interventions were conceptualised as either: interventions incorporating components delivered via computer, tablet, website, e-mail, or mobile app; or interventions delivered via text messaging, telephone calls, or telehealth/videoconferencing modalities.

### Search strategy

Medline, Embase, CINAHL and Cochrane Library of Critical Reviews electronic databases were searched from 1990 to Dec 2016 using subject headings and keywords (Additional file 1). The reference lists of existing systematic reviews and all eligible intervention studies were manually searched.

### Inclusion/exclusion criteria

Studies were included if they: (i) examined interventions as defined above; (ii) included people providing unpaid/informal support to someone living with dementia (aged 18 years or over); (iii) examined one or more of the following outcome(s): health-related effects (e.g. impact of caring for someone living with dementia, quality of life, depression, satisfaction with care, self-efficacy), health care utilisation and/or costs (e.g. hospital admission, length of stay). Studies with a heterogeneous sample were included if they reported outcomes separately for caregivers. Studies were excluded if they: (i) were reviews, case studies, commentaries, conference abstracts, editorials or protocol papers; (ii) tested interventions that were not caregiver–oriented (e.g. education of professionals).

### Data coding

The abstract and title of retrieved articles were initially assessed against the eligibility criteria by one reviewer and rejected if the study did not meet inclusion criteria. The remaining full-text studies were assessed against the inclusion criteria by one author, and studies which met all criteria were retained. A random sample of 20% was coded independently by another author. Discrepancies between the two authors were resolved through discussion. Studies were categorised as either measurement, descriptive or intervention. ***Measurement studies*** reported on the development or psychometric properties of tools to assess utilisation and/or acceptability. ***Descriptive studies*** reported on perceived acceptability, access or utilisation of interventions. ***Intervention studies*** testing the effectiveness of interventions that had a primary aim of improving health outcomes, health care utilisation or costs for caregivers of people with dementia.

### Assessment of methodological quality

Intervention studies were assessed to determine whether the experimental design was one of the four types allowed by the EPOC design criteria - randomized controlled trials, controlled trials, controlled before and after studies, or interrupted time series studies [21]. Stepped wedge designs were also included as they are a viable alternative to a parallel cluster randomised trial and accepted as a robust design. For those studies meeting minimum design criteria, methodological quality was then assessed using EPOC risk of bias criteria independently by two reviewers (AW and SD).

### Assessment of intervention effectiveness

Study data was extracted for those studies that met EPOC criteria by authors (AW, SD, EM) and included: sample characteristics, intervention details, outcome measures and time points when data were collected, intervention effects on primary and secondary outcomes, study limitations and conclusions. Study data on feasibility and acceptability, including: retention of participants; access to the internet; engagement strategies; and utilisation of technologies was also reported.

### Analysis

Poisson regression was used to model trends over time in the numbers of publications. Percent change by year is presented and *P*-values were calculated from the Wald Chi-square.

## Results

### Search results

**Fig. 1:**
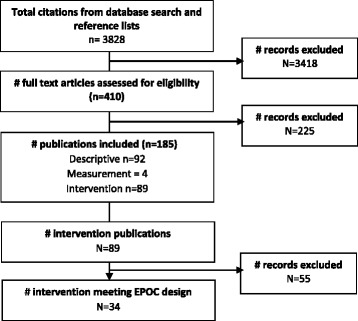
Search strategy

A flow diagram of the search strategy is provided in Fig. 1. After duplicates were removed, 3828 publications were identified and assessed against the eligibility criteria. A total of 185 publications met eligibility criteria and were included.

### Number and type of studies published between Jan 1990 and Dec 2016

**Fig. 2:**
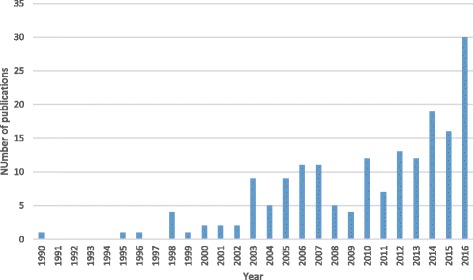
Number of data-based publications by year January 1990–December 2016

Half of the eligible studies were descriptive studies (*n* = 92; 50%) (see Fig. 2). Four measurement studies were identified, which tested the validity and reliability of instruments to assess the utilization or acceptability of interventions. The remaining 89 studies (48%) reported on interventions to improve the health outcomes of caregivers. Poisson regression shows evidence of the number of publications increasing by 13% each year (*p* < 0.001).

### Methodological quality of intervention studies

Of the 89 intervention studies identified, only 34 met initial EPOC design criteria (45%). These included a cluster randomised controlled trial, 30 RCTs, and four non-randomised controlled trials (see Table 1). The 34 studies were rated across each of the nine EPOC risk of bias criteria. Only two studies were low risk on all of the nine criteria; with ten studies rated as low risk on at least seven of the criteria. The most poorly met criteria included: not specifying method of generating allocation sequence or concealing allocation, and blinded outcomes assessment.

**Table 1:** Quality of intervention studies meeting EPOC methodological criteria (Low, High, Unclear)

Author, Date,	Study type	Allocation sequence adequately generated?	Concealment of allocation	Baseline outcome measurement similar	Baseline characteristics similar	Incomplete outcome data adequately addressed	Knowledge of allocated interventions prevented	Protections against contamination	Selective outcome reporting	Free other risk of bias
Au 2015 [36]	RCT	U	U	L	L	L	L	L	L	L
Au 2014 [37]	RCT	U	U	L	L	L	L	L	L	H
Beauchamp (2005) [23]	RCT	U	U	L	L	L	U	L	L	L
Blom (2015) [28]	RCT	L	L	L	L	L	L	L	L	L
Brennan (1995) [27]	RCT	U	U	L	U	L	U	L	L	L
Chang 1999 [44]	RCT	U	U	L	L	H	H	H	L	L
Connell 2009 [45]	RCT	H	U	L	L	H	U	U	L	H
Cristancho 2015 [30]	RCT	L	U	L	L	L	H	L	L	H
Czaja 2013 [53]	RCT	U	U	L	L	L	L	L	L	L
Davis 2011 [35]	RCT	U	U	H	L	L	L	L	L	L
Esierdorfer 2003 [49]	RCT	U	U	L	U	L	U	L	L	L
Finkel 2007 [50]	RCT	U	U	L	L	H	L	L	L	H
Glueckauf 2007 [41]	RCT	U	U	U	U	H	H	L	L	L
Goodman 1990 [40]	RCT	U	U	U	L	H	H	U	L	L
Hicken 2016 [55]	RCT	U	U	H	U	L	U	U	L	H
Lai 2013 [22]	RCT	U	U	U	H	L	U	U	H	H
Mahoney 2001, 2003 [51, 52]	RCT	L	L	L	L	L	L	L	L	H
Martindale 2013 [39]	RCT	U	U	L	H	L	U	L	L	L
Marziali 2006 [47]	RCT	U	U	L	L	H	H	L	H	L
Marziali 2011 [48]	NRCT	H	H	L	U	L	U	U	L	L
Nunez 2016 [31]	RCT	L	U	L	H	U	U	L	L	L
Pagan-Ortiz 2014 [29]	NRCT	H	H	U	U	U	U	U	L	H
Steffen 2016 [54]	RCT	L	U	L	L	L	U	L	L	L
Torkamani 2014 [26]	RCT	U	U	L	L	H	U	L	L	L
Tremont 2008 [32]	RCT	L	U	L	L	H	L	L	L	L
Tremont 2015 [46]	RCT	L	L	L	L	L	L	L	L	L
van de Roest 2010 [24]	NRCT	H	H	L	H	L	H	L	L	H
Van Mierlo 2012 [38]	NRCT	H	H	L	L	U	H	H	L	L
Van Mierlo 2015 [25]	CRCT	L	L	L	H	L	U	L	L	L
Wilz 2011 [42]	RCT	L	L	L	H	L	L	L	H	L
Wilz 2016 [43]	RCT	L	L	L	H	L	L	L	H	L
Winter 2007 [24]	RCT	U	U	L	H	U	U	U	L	L
Wray 2010 [34]	RCT	U	U	L	L	L	U	L	L	L

### Intervention study characteristics

Table 2 presents the study characteristics of intervention studies that met EPOC design criteria (*n* = 34). Sample sizes ranged from 11 [22] to 299 [23] participants. The majority of the studies were conducted in the United States of America (USA) (*n* = 20), Europe (*n* = 8), China (*n* = 3), Canada (n = 2) and the United Kingdom (UK) (*n* = 1). Ten interventions were delivered by computer only [22–31]; 15 via telephone [32–46]; and nine tested combined multiple modalities, such as computer-telephone systems [47–55]. Almost all adopted psychotherapeutic approaches: behavioural activation [36, 37, 54]; psycho-education alone or combined with cognitive behavioural therapy, skills training and/or peer support [23–32, 35, 38, 39, 50, 53, 55, 56]; peer support groups [33, 40, 47, 48]; CBT [41–44]; or family therapy [49]. One study incorporated physical activity and psychotherapy [45]. Length of follow-up ranged from 1 month to 18 months. Attrition was moderate to high across studies (mean = 23%, range 6% [27]-61% [41]); with differential dropout reported between the intervention group (40%) compared to control (11%) [28]. Some studies did not report rate or reasons for loss to follow-up [22, 24, 26, 33, 34, 38, 48].

**Table 2:** Acceptability, feasibility and effectiveness of interventions in improving outcomes in high quality studies

Reference, Country Design	Sample size; Consent rate, Setting	Eligibility Inclusion/exclusion criteria	Intervention and control characteristics	Outcomes and data collection time points	Results of the study	Acceptability, engagement and utilisation of intervention
Telephone counselling						
Au, Alma 2015 [36] China RCT	Sample: 96 Consent: 81% Setting: Two hospitals	Inclusion criteria: ≥25 yrs.; carer of diagnosed Alzheimer’s Disease for ≥3mths; primary carer and spouse, kin/sibling Exclusion: intellectual deficit; suicidal ideation; psychotic disorder; not fluent in Chinese/Cantonese	Intervention: Telephone based intervention: behavioural activation (8 bi-weekly calls) *Delivered by*: Trained volunteers Control group: Telephone-based intervention: psychoeducation and communication (8 calls)	Primary: Depression (CES-D) Secondary: Use of emotional regulation strategies Follow-up: 1 and 5 months	Significantly decreased levels of depressive symptoms in intervention group Increased use of emotional regulation strategies in intervention group	NR
Au et al. 2014 [37] China RCT	Sample: 60 Consent: 92% Setting: One hospital	Inclusion criteria: ≥25 yrs.; primary full time carer for ≥6mths; spouse or daughter/son Exclusion: intellectual deficit; suicidal ideation; psychotic disorder; not fluent in Chinese/Cantonese	Intervention: Telephone assisted intervention: pleasant event scheduling Control group: Standard care from psychogeriatric team	Primary: Depression (CES-D) Secondary: Self-efficacy (Revised scale for care-giving self-efficacy) Follow-up: 1 and 2 months	Significantly decreased levels of depressive symptoms in intervention group No difference in self-efficacy between the groups Limitations: small sample size; significantly higher baseline depression scores in intervention	NR
Chang et al. 1999 [44] RCT USA	Sample: recruited: 102; analysed: 83 Consent rate: NR Setting: Community, Alzheimer’s Association	Inclusion criteria: spoke English; access to VCR & phone; lived with dementia sufferer who had problems eating and dressing Exclusion: NR.	Intervention: Video and telephone-based: video (20 mins); telephone interviews; problem-solving (bi-weekly for 12 wks) *Delivered by*: Gerontological clinical nurse specialists Control group: attention only: calls made but based on general discussion only	Primary: Burden, satisfaction, anxiety, depression Follow-up: 3 mths	Lower depression in intervention than in control group Decrease in anxiety, and emotion-focused coping strategies over time in both groups No difference in burden between the groups	Viewed tapes once or twice 5–90 min calls for intervention; 5–30 min for control Satisfied with calls
Connell & Janevic 2009 [45] RCT USA	Sample: recruited: 157; analysed @ 6mth: 137; analysed @ 12mth: 130. Consent rate: 47% Setting: Alzheimer’s DRC & Association	Inclusion criteria: primary caregiver for a spouse with dementia; living with spouse at home; interest in increasing physical activity. Exclusion: NR.	Type: Telephone-based (weekly for 2mths; bi-weekly for 2mths; monthly for 2mths): goal-setting; counsellor feedback; self-monitoring *Delivered by*: behaviour-change counsellors Control group: no materials provided during intervention period	Primary: Self-rated physical health; Physical function (MOS SF General Health Survey); caregiver burden (RMBCP); Exercise time; Exercise self-efficacy; Self-efficacy; depressive symptoms (CES-D). Follow-up: 6 and 12 months	At 6 mths follow-up, intervention reported reduced perceived stress At both 6 & 12 mths follow-up intervention reported reported greater exercise self-efficacy	Calls at participant convenience 16% loss to follow-up Acceptability NR
Davis et al. 2011 [35] RCT USA	Sample: recruited: 53; analysis: 46 Consent rate: Setting: nursing home	Inclusion criteria: ≥18 yrs.; care recipient in NH in ≤2 mths; caregiver; ≥4 h /day caring in 6 mths; Exclusion:	Intervention: One initial care, with 7 weekly follow-up calls, and 2 biweekly termination calls over the third month *Delivered by:* Master’s level therapist Control: usual care	Primary: Guilt; depression (CES-D); burden (ZBI), hassles with NH staff (Nursing Home Hassles Scale); satisfaction (ODAFSI); Follow-up: 3 months	Intervention participants reported greater reduction in feelings of guilt, and fewer problems and concerns with NH care. No benefit of intervention for depression or burden.	Attrition 13% Highly satisfied with service and treatment, would use again
Glueckauf et al. 2007 [41] RCT USA	Sample: recruited: 36; analysed: 14 Consent rate: NR Setting: Rural area of Florida	Inclusion criteria: ≥6 h p/wk. caring for ≥6mths; short term problems amenable to a short-term intervention; no difficulties hearing over phone; CR has ≥1 limitation in basic activities of daily living; 2 dependencies in instrumental activities associated with daily living Exclusion: CG terminal illness; CR life expectancy <6mths; severe illness other than dementia; psychological problems	Intervention: Phone CBT: 5 x weekly individual session; 7 x weekly group session; goal-setting; self-monitoring *Delivered by*: trained doctoral or master’s-level counsellor Control group: Written education material and toll-free telephone line if needed	Primary: Subjective burden (CAI) Secondary: Caregiving self-efficacy (CSES); depression (CES-D); problem change measures (ISS; IFS; ICS); Treatment satisfaction (CSQ-8) Follow-up: 3 mths	No sig differences between the groups in burden; trends towards improvements in both groups Intervention group reported trend towards reduced depressive symptoms	Guide and training Moderate to high satisfaction
Martindale-Adams et al. 2013 [39] RCT USA	Sample: recruited and analysed: 154 Consent rate: 70% Setting: VA hospital	Inclusion criteria: Family members reporting stress or difficulty with care; living with care recipient; ≥4 h care or supervision per day for ≥6mths; care recipient has dementia or MMSE ≤ 23; ≥1 ADL or 2 IADL limitations; ≥1 member of dyad as veteran services from VAMC Exclusion: Planned nursing home admission ≤6mths	Intervention: Telephone-based (bi-weekly for 2mth; monthly thereafter for 1 yr): support groups; education; skills building; caregiver notebook *Delivered by*: master’s-prepared group leaders Control group: Pamphlets and phone numbers of local resources	Primary: Social support (items re. received support, satisfaction, social networks) Secondary: Health (SF-36); self-care (REACHII questions); Burden (Zarit); depression (CES-D); general well-being (General Well-Being Scale) Bother (RMBPC). Follow-up: 6 and 12 mths	No significant benefit of the intervention on any outcome	61% completed ¾ sessions, 77% half sessions and 8% < 3 sessions Intervention helpful Valued different perspectives, support and interaction
Tremont et al. 2008 [32] RCT USA	Sample: recruited: 60; analysed: 33 Consent rate: NR Setting: Southern New England region of USA	Inclusion criteria: carer: ≥21 years; lived with relative with Dementia; ≥4 h p/day care ≥6mths; care recipient: formal Dementia diagnosis; CDR 1 or 2; ≥50 yrs. Exclusion criteria: Carer had psychiatric illness or cognitive impairment	Intervention: telephone-based: 23 calls across 1 yr.; therapist contact; individualised *Delivered by:* Master’s level therapist Control group: Usual care	Primary: Depression (GDS); Caregiver burden (ZBI); Reaction to memory and behaviour problems (RMBPC). Secondary: Alzheimer’s Disease Knowledge Test; SF36 General Health; Family Assessment Device; Multidimensional Scale of Perceived Social Support. Follow-up: 1 yr	Intervention group reported significantly lower burden scores Intervention group reported less severe reactions to memory and behaviour problems.	Satisfied with service (94%); met needs (77%); recommend to friend (88%); satisfied with therapist skills (100%); convenience (94%), written materials (88%), and clear (94%); overall (94%). Calls <30 min
Tremont et al. 2015 [46] RCT USA	Sample: recruited: 250; analysed: 212 Consent rate: 84% Setting: hospital and community based	Inclusion criteria: carer: ≥2 negative caring experiences; Care recipient: formal Dementia diagnosis; living in community; no planned placement in care in next 6mths. Exclusion criteria: Carer: major acute illness; not English speaking; cognitive impairment; care ≥6mth for ≥4 h supervision p/day; no telephone access	Family Intervention: Telephone Tracking—Caregiver (FITT-C): 16 calls across 6mths; psycho-education; self-assessment + summary letter *Delivered by:* Master’s level therapist Intervention 2: Telephone-based: 16 calls, non-directive support via active listening and open questions *Delivered by:* Master’s level therapist	Primary: Depression (GDS); Caregiver burden (Zarit); Reaction to memory and behaviour problems (RMBPC). Secondary: Family Assessment Device (FAD); Self-efficacy (SEQ); positive aspects of caring (PAC); Health related QoL (Euro-QoL). Follow-up: 6 mths	Intervention group reported significantly improved caregiver depressive symptoms and significantly reduced reactions to care-recipient depressive behaviours.	Intervention perceived program more logical and likely to reduce burden than control Both groups satisfied Average 1.81 missed calls for intervention and 1.22 for control Average call: 37 mins intervention; 30 mins for control
Van Mierlo et al. 2012 [38] CBA The Netherlands	Sample: recruited: 54 Consent rate: NR Setting: Amersfoort-Leusden, Utrecht, Amsterdam, and Laren and Huizen	Inclusion criteria: Informal caregivers of people with Dementia living at home Exclusion criteria: NR	Intervention 1: telephone-based coaching only: 10 × 30 min call every 2-3wks; coaching *Delivered by:* health professional trained coaches Intervention 2: telephone-based coaching with respite care: 10 × 30 min call every 2-3wks; coaching; (no description of respite given) *Delivered by:* health professional trained coaches Control group: Respite care only	Primary: Burden (SSCQ); mental health problems (GHQ-28) Follow-up: 20 wks	Telephone plus respite participants reported significantly less burden than telephone-only participants. Telephone plus respite participants reported significantly fewer mental health problems than control.	Caregivers valued the telephone intervention and were generally satisfied with it. Coaches participated in an average of 7.6 sessions with caregivers
Wilz et al. 2011 [42] RCT Germany	Sample: recruited: 229; analysed: 172 Consent rate: 88% Setting: Berlin/Brandenburg and Thuringia	Inclusion criteria: full time carer; care recipient diagnosis of dementia; GDS score > 3 Exclusion criteria: Simultaneous psychotherapy; cognitive impairment; severe acute mental/physical condition; care recipient cared for in day care >3 days p/wk	Intervention: telephone-based: 7 x session 3 mths; CBT; structured with some flexibility for individualisation *Delivered by:* CBT-trained clinical therapists Control group 1: Progressive Muscle Relaxation in same conditions as experimental gp; written material & CD for PMR *Delivered by:* PMR psychologists Control group 2: untreated	Primary: Goal attainment (GAS) Secondary: None Follow-up: 6mths	Overall: 30.1% (*N* = 25) of the participants achieved complete goal attainment, 39.8% (*N* = 33) reached partial attainment, and 24.1% (*N* = 20) declared no change Participant goals mostly matched intervention strategies	2/3 both groups program very good; 1/3 good. 7 sessions not enough; control felt it was enough or too much 81% very helpful 72% felt expectations fulfilled Significant difference between intervention and treated control in duration of sessions
Wilz & Soellner 2015 [43] RCT Germany	Sample: recruited: 229; T1: 191; T2: 182 Consent rate: 94% Setting: Berlin/Brandenburg and Thuringia	Inclusion criteria: full time carer; care recipient diagnosis of Alzheimer’s disease; GDS score > 3 Exclusion criteria: Simultaneous psychotherapy; cognitive impairment; severe acute mental/physical condition; care recipient cared for in day care >3 days p/wk	Intervention: telephone-based: 7 × 60 min session; CBT; multi-component; individualised *Delivered by:* CBT-trained clinical therapists Control group 1: attention control: telephone-based Progressive Muscle Relaxation *Delivered by:* PMR psychologists Control group 2: untreated control	Primary: Perceived body complaints (GBB-24); emotional wellbeing & perceived health status (VAS) Secondary: None Follow-up: 3 & 6 mths	T1: Significantly higher perceived health status in CBT group compared to untreated. Significant increase in depressive symptoms in PMR group compared to CBT group T2: Significant improvements in emotional wellbeing and body complaints in CBT group CBT group improved in emotional wellbeing whereas PMR group decreased in emotional wellbeing Exhaustion significantly decreased in CBT group whereas increased in untreated control	Very good (71.9%) and good (27%) 90.9% recommend to others 81% very helpful 71.8% completely fulfilled expectations Except for dropouts, all in intervention group completed all 7 sessions No interruptions in treatment: 85.5%; control: 79.4%
Telephone support group						
Goodman and Pynoos 1990 [40] RCT USA	Sample: recruited: 81; analysed: 66 Consent rate: NR Setting: Community	Inclusion criteria: NR Exclusion: NR	Intervention: Telephone-based: peer telephone networks; 4–5 caregivers in each network; members of network called one another rotating across 12 week period *Delivered by:* peers Control group: lecture: 12 x telephone access lectures about Alzheimer’s disease	Primary: Problems (Memory & Behaviour Problem Checklist); Burden (Burden Interview Caregiver Elder Relationship Scale); mental health (scale by Rand Institute); social support (network measure adapted from Vaux & Harrison; perceived social support caregiving); knowledge Secondary: NR Follow-up: 3 mths	Intervention participants reported significantly higher perceived social support and information gains. Trends towards improved satisfaction with caregiving among intervention participants.	Bi-monthly follow-up calls Acceptability and utilisation NR
Winter & Gitlin 2006 [33] RCT USA	Sample: recruited: 103 Consent rate: NR Setting: NR	Inclusion criteria: female; ≥50 yrs.; ≥6mths care to relative with diagnosis of ADRD; weekly telephone access ≥ 1 h Exclusion criteria: NR	Intervention: telephone-support groups: hourly weekly session with 1 x facilitator 5 x caregiver; problem-solving; education *Delivered by:* Trained social workers Control: usual care	Primary: Caregiver depression (CES-D); burden (ZBI); perceived personal gain (Gain Through Group Involvement Scale). Secondary: None Follow-up: 6 mths	No significant difference on outcomes between the groups	Attendance not associated depression, burden or gains Wives, older and African Americans participated in more sessions. Average of 14.8/26 sessions in 6mth.
Wray et al. 2010 [34] RCT USA	Sample: recruited: 158 Consent rate: 33% Setting: New York Veteran Affairs Network	Inclusion criteria: caregiver: primary caregiver; lived with veteran ≥1 yr.; caregiver strain index ≥7. Care recipient: lived in own home; dementia diagnosis; spouse/partner living with them ≥1 yr.; GDS ≥ 3or dependent on ≥1 activity of daily living & ≥ 3 instrumental ADLs, Exclusion criteria: caregiver participating in other support group; caregivers not spouses	Intervention: telephone-based: ≥8 caregivers during 10 weekly sessions; no video conferencing; homework; education; coping; group support *Delivered by:* Three master’s-prepared social workers and one nurse dementia care manager Control: usual care	Primary: Healthcare cost (inpatient; outpatient; nursing home; pharmacy costs) and utilisation (total bed days of care; total admissions; total visits) Secondary: None Follow-up: 6 & 12 mths	Total health care costs significantly lower in intervention group compared to control group at 6 mths, but not at 12 mths. No significant interactions in utilisation over time.	NR
Computer-based						
Beauchamp et al. 2005 [23] RCT USA	Sample: 299 Consent: NR Setting: Online	Inclusion criteria: ≥part time employment; ≥4 contacts a mth caring for a family member with memory problems; reports of stress from caregiving Exclusion: NR	Intervention Web-based: text; videos; links to tailored content; modules (available for 30 days) Knowledge, behavioural and cognitive-based skills Control group: Waitlist control	Primary: Depression(CES-D), anxiety (STAI), caregiver strain (Caregiver Strain Scale); stress Secondary: Self-efficacy (6 questions); coping skills (Revised Ways of Coping) Follow-up: 1 month	Intervention group reported significant improvements in depression, anxiety, level and frequency of stress, caregiver strain, self-efficacy, and intention to seek help, as well as perceptions of positive aspects of caregiving. Those who viewed the program more had greatest benefit	59% used once, 19% twice, 11% 3 times, 11$ 4+ times; 32 mins average; dose-response relationship Email reminder to non-users Acceptability survey
Blom et al. 2015 RCT [28] Netherlands	Sample: recruited: 245; analysed: 175 Consent rate: NR Setting: Online	Inclusion criteria: family caregivers; some symptoms of depression / anxiety / feelings of burden (CES-D > 4 or HADS-A > 3 or a burden score of at least 6 on a scale ranging from 0 to 10). Exclusion: NR.	Intervention Web-based: lessons, coaching, feedback, homework, text, videos, exercises *Coaching component delivered by:* psychologist trained in CBT Control group: Digital newsletters: information only; no coaching contact	Primary: Depression(CES-D) Secondary: Anxiety (HADS) Additional: Functional status of dementia patient (IQCODE); Perceptions of distress (Self-perceived pressure from informal care); Caregivers distress (RMBPC); Competence (SSCQ); Sense of mastery (Abb Pearlin Mastery Scale) Follow-up: 3 and 6 months	Significantly lower depression and anxiety in intervention group compared to control group	Higher drop out in intervention arm (40% vs 11%) Engagement and acceptability NR
Brennan et al. 1995 [27] RCT USA	Sample: recruited: 102; analysed: 96 Consent NR Setting: Online	Inclusion criteria: primary family caregiver for person with Alzheimer’s disease at home; local telephone exchange; read and write English Exclusion: NR.	Intervention Web-based: questions to guide decision-making; public and private peer communication Control group: placebo training identifying local services and resources	Primary: Decision-making confidence (modified decision confidence scale); decision-making skill (investigator-developed self-report) Secondary: Social Support (IESS); Burden (Impact of caregiving scale); Depression (CES-D); contact with services Follow-up: 1 year	Enhanced decision-making confidence in intervention group Decision-making skill unaffected	Training, monthly phone calls on use Mean access 83 times 13 mins average use Communication component used most
Cristancho-Lacroix et al. 2015 [30] Pilot RCT France	Sample: 49 Consent rate: 55% Setting: Day care center geriatric unit	Inclusion criteria: ≥18 yrs.; French speaking; caregiver for community-dwelling person with Alzheimer’s; met criteria for DSM of mental disorders; ≥4 h with relative; ≥12 PSS-14; internet access. Exclusion: Professional caregivers	Intervention: web-based; thematic sessions; weekly release of sessions; text; video Control group: usual care	Primary: Perceived stress of caregivers (PSS-14) Secondary: Self-efficacy (RSCS); Perception and reaction to symptoms (RMBCP); Subjective burden (ZBI); Depression (BDI-II); Self-perceived health (NHP) Follow-up: 3 and 6 months	No effect on self-perceived stress 3mths, however the intervention improved knowledge of illness	Training and user manual provided 71% finished protocol Use average 19.7 times; for 262 min Useful, clear and comprehensive
Lai et al. 2013 [22] Pilot RCT China	Sample: recruited: 11; analysed: 11 Consent rate: NR Setting: Online and offline (no indication of place of recruitment)	Inclusion criteria: primary caregiver; read and write Chinese Exclusion: Domestic helper; already using online support group; caring for others; care recipient requires total care	Intervention: Web-based: training workshops; forum Control group: Onsite workshop delivered by social workers or nurses	Primary: Depression (CES-D) Secondary: General Health (GHQ-30); Alzheimer’s Disease Knowledge; Caregiver burden (ZBI); QoL (WHO QoL Measure – Brief) Follow-up: 7 wks	Intervention participants reported greater knowledge gained Control group participants anxiety and depression dropped significantly after the workshop	Utilisation, engagement not reported Convenient
Nunez-Naveira et al. 2016 [31] RCT Spain	Sample: 77 recruited, 61 analysed Consent rate: NR Setting: Non-profit organisations, geriatric clinic	Inclusion: primary carer of someone with GDS 4 or more; basic care tasks for a minimum of 6 weeks, no remuneration ZBI score above 24 Exclusion criteria cognitive impairment, illiterate, severe hearing, visual, motor problems	Intervention Learning section with information on 15 topics; Daily Task and Social Networking. Control group: did not use the application	Primary: Depression (CES-D) Follow-up: 3 mth (post-intervention)	Intervention group reported statistically significant fewer depressive symptoms pre- versus post-intervention (*p* = 0.037). No difference for control.	Non-completion rate 20% Technical, pedagogical and general satisfaction lowest scores for Smartphone users
Pagán-Ortiz et al. 2014 [29] NRCT USA	Sample: recruited: 72; T1: 40; T2: 32 Consent rate: NR Setting: Cities	Inclusion criteria: Spanish speaking caregivers of Dementia sufferers Exclusion criteria: NR	Intervention: web-based: limited text crowding; carer photos; 4 × 1.5 h group sessions Control group: Printed educational materials	Primary: Mastery and confidence (PMS); social support (LSNS); burden (ZBI); emotional distress (CES-D) Follow-up: 1 mth	Across all outcomes there was a trend towards improvements in intervention group but this was not significant	Training Visit time 30mins-1 h average Majority visited >3 times Beneficial, better for early stages, would recommend
Torkamani et al. 2014 [26] RCT UK	Sample: recruited: 30 Consent rate: NR Setting: Three European hospitals	Target: Caregivers and people with dementia Inclusion criteria: Patient living at home with full time carer; BI score ≥ 35; ≥9 MMSE <21; Dementia as primary condition or as Parkinsons Disease. Exclusion criteria: NR	Intervention: web-based: education; music; relaxation techniques; forum; self-monitoring tasks. Control group: Usual care	Primary (Carer): Burden (Zarit); Psychiatric/behavioural problems (NPI); Depression (Behavioural: DBI; Sensory: Zung); Quality of life (EQ5D;). Primary (PwC): Cognitive functioning (MMSE; DRS2); everyday activities, self-care and personality change (BDRS); Clinical Dementia Rating Scale; memory and behaviour (RMBPC); Depression (GDS); Functional disability (BI); daily living (LADL); comorbidity (CCI) Follow-up: 3 & 6 mths	Significant improvement in QoL of caregivers in intervention participants, with some reduction in burden and distress.	Training in program Confidence and awareness of health, provided dementia information
Van der Roest et al. 2009 [24] NRCT Netherlands	Sample: recruited: 28 Consent rate: NR Setting: Amsterdam Diagnostic group: Caregivers and people with dementia	Inclusion criteria: general: ≥4 h per/week caring for community-dwelling dementia patient; to be in experimental: care recipient lives in Amsterdam district; familiar with computers and the internet Exclusion criteria: NR	Intervention: internet-based: tailored; information; resources; advice Control group: Usual care	Primary: Needs assessment (CANE); burden (SSCQ); self-efficacy (PMS) Secondary: QoL (QoL-AD); knowledge about care and welfare Follow-up: 2 mths	Intervention participants reported more met and less unmet needs, and higher competence	Intervention was easy to learn and relatively user friendly Intervention used program 5.14 times Mean session duration: 14:36 mins
Van Mierlo et al. 2015 [25] RCT The Netherlands	Sample: recruited: 73; analysed T1: 64; analysed T2: 49 Consent rate: 89% Setting: Several regions of the Netherlands (Amsterdam Zuidoost, Amsterdam Nieuw-West, regions of Lelystad and Amstelveen)	Inclusion criteria: Informal caregivers of people with Dementia; computer with internet capabilities; knows how to use computer Exclusion criteria: Not able to understand/read Dutch; anticipated nursing home admission ≤6mths	Intervention: internet-based: available for 1 yr.; case manager and carer access tailored; advice Control group: Usual care	Primary: Needs of people with Dementia (CANE) Secondary: Competence (SSCQ); QoL (EQ5D + c); stress (NPI) Follow-up: 6 mths; 12 mths	Increased competence in intervention participants at 12 mths. Active users in the intervention group reported more met needs than controls at 6 mths.	Easy to learn and user friendly. 5 pts. never logged in. 5 in intervention never logged in 16 classified as low frequent users (≤6 logins); 20 classified as high frequent users (≥7 logins).
Multi-modal						
Czaja et al. 2013 [53] RCT USA	Sample: recruited 110 Consent rate: 87%	Inclusion criteria: ≥21 yrs.; English/Spanish speaking; caregiver for person with AD; ≥4 h /day caring in last 6 mths; MMSE patients < 24; telephone. Exclusion: carer/PWD illness, MMSE = 0	Intervention: education and skills training, 6 × 1-h sessions (2 in-home and 4 via video); 5 video support groups. *Delivered by:* online and certified interventionists Attention control: same level of contact - nutrition and diet. Control group: written education materials + brief phone call.	Primary: Depression (CES-D); Revised Memory and Behavior Problems Checklist; social support; positive caring. Follow-up: 5 month follow-up	Intervention participants experienced decreases in caregiver burden, increased appreciation of the positive aspects of caregiving, and greater satisfaction with social support. No benefit of the intervention for depression.	Useful, easy to use, support groups, video-phone and resource guide valuable
Eisdorfer et al. 2003 [49] RCT USA	Sample: recruited: 225; analysed: 147 Consent rate: NR Setting: Miami site of REACH program	Inclusion criteria: care recipient had probable ADRD or MMSE < 24; care recipient has dependency/limitation in daily living; carer lives with patient; ≥4 h per day caring for ≥6 mths; one other family member agrees to participate who provides emotional/instrumental support. Exclusion: Caregiver has a terminal/severe illness/disability; not residing in Miami in 6mths.	Intervention 1: computer-telephone integrated system: calls; discussion groups; voice messaging; therapist reminders; resources Intervention 2: Structured family therapy (SET) *Delivered by:* therapists Control group 2: Minimal therapy	Primary: Depression (CES-D) Secondary: Caregiver burden (RMBPC); Satisfaction with social support Follow-up: 6 and 18 months	At the 6 mths follow-up the integrated system group reported significant reduction in depressive symptoms, relative to other groups At 18mths follow-up the reduced depressive symptoms was maintained for Cuban Americans and White Non-Hispanics	User guides, reminders 56 average contacts 19 h average time using system
Finkel et al. 2007 [50] Pilot RCT USA	Sample: recruited: 46; analysed: 36 Consent rate: NR Setting: Community-based social service agency	Inclusion criteria: ≥4 h care per day for a relative with Alzheimer’s or dementia for ≥6mths; ≥21 yrs.; living with or same geographic area as patient; telephone; intending to stay in geographic area ≥ 6mths; English competency; MMSE ≤ 23 Exclusion: Caregiver or care recipient has: life expectancy ˂6mths; blind or deaf. Care recipient MMSE = 0 or bedbound.	Intervention: computer-telephone integrated system: calls; messaging; information and services; education sessions; support group sessions. 2 x in-home sessions (first & @ 6mths) *Delivered by*: certified clinical social workers Control group: Basic education material; 2 x call <15mins	Primary: Depression (CES-D) Secondary: Caregiver burden (RMBPC); Caregiver health care behaviours (Caregiver Health & Health Behaviour Scale); Social Support (Revised scale of Inventory of Social Supportive Behaviors) Follow-up: 6 mths	Intervention participants reported significantly reduced burden Intervention participants with high depression at baseline reported significant decline in depression Intervention participants reported significantly increased confidence in caregiving and improved ability to provide care	Trained in use 60% completed all sessions 80% support groups attendance 8 h contact over study Support groups valuable, system easy to use, helpful, valuable
Hicken et al. 2016 [55] RCT USA	Sample: 231 caregivers; stratified by level comfort with internet and rurality. Consent rate: NR Setting: VA medical centre, residing in community	Exclusion: care recipient bedbound; had cancer or serious mental illness diagnosis; life expectancy of <16 weeks; unable to give informed consent	Intervention 1: Internet via computer 3 days per week for 10–15 min; videos caregiving skills; written information; brief health assessments 2–3 per week; *Remote monitoring* by Case Manager Intervention 2: Telephone support printed materials, DVD; monthly telephone calls; *Monitoring by* Case Manager	Primary: Caregiver burden (ZBI); Grief (MARWIT); Depression (PHQ); family conflict (2 items); nursing home placement (DIS). Follow-up: Baseline and post-intervention (4–6 mths)	No differences between groups on depression, burden, nursing home placement or family conflict. For experienced internet users greater reduction in grief was reported those receiving internet vs increase in symptoms for telephone.	74/231 not comfortable with internet at baseline Interacting with Case Manager important support
Mahoney 2001 [52] RCT USA	Sample: recruited: 100; analysed T1: 93; analysed T2: 86; analysed T3: 82 Consent rate: 85% Setting: Visited in their homes	Inclusion criteria: >21 yrs.; ≥4 h daily assistance ≥6mths for family member with AD with ≥2 IDL impairments and ≥AD-related disturbing behaviour. Exclusion: Plan to institutionalise family member ≤6mths; participating in other study; terminally ill.	Intervention Integrated telephone-computer system: modules; mailbox; voice messaging; bulletin board. Control group: Usual care	Primary: Bothersome nature of caregiving (RMBPC); Anxiety (STAI); Depression (CES-D) Follow-up: 6, 12, 18 mths	Adopters were older higher education and greater sense of management Those judged as more highly proficient at study commencement by RA were more likely to be adopters Preferred human interactions Effectiveness see Mahoney 2003	Training in system 55 min per user 50% at least 22 mins, 25% at least 70 mins 21% ask the expert, 24% in home support group, 57% respite and 79% counselling Use plateau first 4 mths, technical issues
Mahoney et al. 2003 [51] RCT USA	Sample: recruited: 100; analysed T1: 93; analysed T2: 86; analysed T3: 82 Consent rate: 85% Setting: Visited in their homes	Inclusion criteria: >21 yrs.; ≥4 h daily assistance ≥6mths for family member with AD with ≥2 IDL impairments and ≥AD-related disturbing behaviour. Exclusion: Plan to institutionalise family member ≤6mths; participating in other study; terminally ill.	Intervention: Integrated telephone-computer system: modules; mailbox; voice messaging; bulletin board. Control group: Usual care	Primary: Bothersome nature of caregiving (RMBPC); Anxiety (STAI); Depression (CES-D) Follow-up: 6, 12, 18 mths	Significant effect on all 3 outcomes for those with lower mastery at baseline Intervention participants (wives only) reported significantly reduced bother	Reminders about features Used most in 4 mths 55 min/user over study 1–45 calls, up to 3 min Preferred short interactions
Marziali & Donahue 2006 [47] Pilot RCT Canada	Sample: recruited: 66; analysed: 48 Consent rate: NR Setting: Two remote hospitals Diagnostic group: Caregivers	Inclusion criteria: Family caregiver; recipient has moderate disability from either Alzheimer’s, stroke-related Dementia or Parkinson’s. Exclusion criteria: NR	Intervention: Internet video-conferencing: 10 × 1 h video support groups; 12 x online support groups; disease-specific support and education *Delivered by:* Group therapists then peers Control group: Usual care	Primary: Health Status (Health Status Questionnaire 12); Depression (CES-D); ADL and IADL; Distress (RMBPC); Social Support (Multi-dimensional scale of perceived social support). Follow-up: 6 mths	No difference between the groups at follow-up on any measure When stress scores were combined (ADL/IADL & RMBPC), a significant effect was found from baseline to follow-up for intervention condition	Training in program 78% easy to use 95% support group via computer positive 61% video-conferencing helpful
Marziali & Garcia 2011 [48] CBA Study Canada	Sample: recruited: 91 Consent rate: NR Setting: Three cities Diagnostic group: Caregivers	Inclusion criteria: Dementia caregivers, spousal or adult children living with care recipient Exclusion criteria: NR	Intervention 1: web-based/video-conferencing: information; email link; chat forum; educational videos; video-conferencing link Intervention 2: web-based: information; educational videos chat forum - clinician moderator	Primary: Caregiver health (HSQ-12); depression (CES-D); caregiver distress (SMAF); current service use; intent to continue caregiving at home Caregiver characteristics: personality (EQO-R); neuroticism; self-efficacy (Revised scale for care-giver self-efficacy) Follow-up: 6 mths	Video-conferencing intervention participants reported significantly great improvements in mental health For video-conferencing participants, improved mental health was associated with lower stress response	Training and facilitated chat forum monthly Video group – average 7 sessions and 5 self-help Education videos not accessed by many Problems with video software
Steffen 2016 [54] RCT USA	Sample: 74 Consent rate: 71% Setting: Primary care Target: Carer and person with dementia	Inclusion: *Carer*: Female 30+; cohabitating with NCD; ≥2 symptoms memory/behaviour; 3 symptoms CES-D; no placement in next 6 months; no suicide attempts or risky alcohol intake; primary care. *Person dementia*: confirmed diagnosis; primary care; no history schizophrenia; bipolar; alcohol, HIV, MS, Korsakoff.	Intervention 1: Behavioural coaching, with videos, workbook, 10 weekly telephone calls and w2 maintenance calls (40 min duration). *Delivered by:* telephone calls from a trained coach Intervention 2: Basic education and support materials +7 telephone calls (20 min duration) *Delivered by:* a trained coach	Primary: Memory and behaviour (RMPBC); depression (BDI-II) Secondary: Mood, Anxiety (MAACL-R), Self-efficacy (RSCS) Follow-up: Intake, post-intervention and 6 months follow-up	Intervention participants reported Greater reduction in: depressive symptoms in intervention (Cohens d = 0.5) and upset due to behaviour in intervention (Cohens d = 0.5). Greater proportion in control had clinically significant depression (53% vs 29%, *p* < 0.05) Lower levels of mood (d = 0.66) and anxiety (d = 0.39) and higher levels of self-efficacy (d = 0.55 and 0.46) in intervention. Benefits for intervention maintained at 6 months	85% completed intervention phase No information on acceptability, engagement or uptake.

*BDI-II* Brief Depression Inventory, *BDRS* Blessed Dementia Rating Scale, *CANE* Camberwell Assessment of Need for the Elderly’, *CES-D* Center for Epidemiologic Studies–Depression Scale, *CCI* Charlson’s Comorbidity Index, *CSQ-8* Client Satisfaction Questionnaire, *EQO-R* Eysenck Personality Questionnaire Revised, *FAD* Family Assessment Device, *GAS* General Anxiety Scale, *GBB-24* Giessen Subjective Complaints List, *GDS* Geriatric Depression Scale, *GHQ-28* General Health Questionnaire – 28 item, *HADs* Hospital Anxiety Depression Scale, *IESS* Instrumental Expressive Social Support Scale, *IQCODE* Informant Questionnaire on Cognitive Decline in the Elderly, *LADL* LAwson Activities of Daily Living; Scale, *LSNS* Lubben Social Network Scale, *MARWIT* MARWIT-MEUSER CAREGIVER GRIEF INVENTORY, *MAACL-R* Multiple Affect Adjective Checklist, *MOS* Medical Outcomes Study, *MSPSS* Multidimensional Scale of Perceived Social Support, *NAS* Negative Affect Schedule, *NPI* Neuropsychiatric Inventory *NR* not reported, *ODAFSI* Ohio Department of Aging Family Satisfaction Instrument, *PAC* Positive Aspects of Caregiving, *PANAS* Positive and Negative Affect Schedule, *PHQ* Patient Health Questionnaire, *PMS* Pearlin Mastery Scale, *PSS-14* Perceived Stress Scale, *QoL-AD* Quality of Life in Alzheimer’s Disease, *RMBCP* Revised Memory and Behavioral Problem Checklist, *RSCS* Reading Self-Concept Scale, *SEQ* Revised Scale for Caregiving Self-Efficacy, *SF-36* Short Form-36, *SMAF* Functional Autonomy Measurement System, *SPICC* Self-Perceived Pressure from Informal Care, *SSCQ* Short Sense of Competence Questionnaire, *STAI* State Trait Anxiety Inventory, *Vas* visual analogue scale, *ZBI* Zarit Burden Interview

### Effectiveness, acceptability and utilization of technology interventions

#### Computer-based interventions

Ten interventions were delivered by computer only [22–31]. Benefits were reported in relation to caregiver depression and mental health outcomes in 3/8 studies [23, 26, 28, 31]; and caregiver burden and/or stress (2/7 studies) (Additional file 2: Table of outcomes) [23, 26]. Other reported benefits included improved knowledge [22, 30], quality of life [26] and unmet needs and more positive aspects of caring [23, 24, 29]. Web-based multimedia interventions providing written and audio-visual materials modelling positive caregiving strategies [23]; a program combined with coach monitoring [28]; and an online platform of educational material combined with peer and clinician contact [26] had modest success. The majority were judged as acceptable, easy to learn and user-friendly by caregivers. However, utilisation was variable. Two studies reported a dose-response relationship, with active users reporting greater benefits [23, 25]. Three studies incorporated online technical support to encourage engagement [22, 23, 27], while others used automated reminders [23, 28]; follow-up calls [27]; health professional contact via chat functions or forums [25]; and financial incentives [23, 29].

#### Telephone interventions

Twelve studies reported on the impact of telephone counselling using psychoeducation [32, 35, 37, 39, 46]; behavioural activation [36] or CBT [41–43]; alone and when supplemented with videos [45, 57] and respite care [38]. Mixed effects were reported in relation to caregiver depression and mental health outcomes (benefits in 6/12 studies) [32, 36–38, 43, 44]; and caregiver burden and/or stress (benefits in 4/11 studies) [32, 38, 45]. Some reported improvements in goal attainment [42], physical symptoms/ADLs [45] and managing problems such as memory and behaviour [32, 46]. No benefits were found for social support, self-efficacy and health and self-care outcomes. Rate of session participation was high in many studies [32, 35, 38, 42, 43, 45, 46], and participants reported the programs as helpful and convenient [32, 35, 38, 39, 41–44, 46]. Four studies reported that the intervention group received longer interventions than their attention-only control groups [42–44, 46].

Three studies examined the impact of telephone support groups. In one of these studies, support groups led to short-term cost savings for veteran care recipients living at home, but this was not maintained over 6 month period [34]. For the other two studies, no significant improvement in the impact of supporting a person with dementia, depression, social support or distress with relative’s problems were identified [33, 40]. The lack of effect did not differ by attendance [33]. Acceptability and utilisation data were not reported in two studies [34, 40].

#### Multi-modal interventions

Nine studies described multi-modal intervention programs. Four of these reported on aspects of the Resources to Enhance Alzheimer’s Caregiver Health for Telephone-Linked Care (REACH) program. A computer-telephone psychoeducational program incorporated home and video-phone delivered family conferencing, online support groups and information resources. In one study, the program resulted in lower burden, and reduced depression for caregivers with elevated baseline depression [50]. However, another found no difference between integrated telephone and computer-network system intervention and control in relation to bother, depression or anxiety at 3 months [51]. In a third study, greater benefits were seen when the program was combined with family therapy [49]. Caregivers who received a psychosocial intervention modelled after REACH II, which included access to support groups via videophone technology, reported a decrease in burden, an increase in perceived social support and positive perceptions of the caregiving experience; but no benefit for depression [53]. Video-conferencing combined with online support led to greater improvements in mental health status compared to an internet chat group alone [48]. Providing the requisite technology or equipment [48, 50–53] and training were seen as an important for promoting uptake and ongoing engagement [48, 50–53]. However, some participants reported problems with using technology [48, 51, 52]. Behavioural coaching including videos, workbooks and telephone calls led to improvements in symptoms of depression, memory and behavioural problems, and caregiver self-efficacy [54]. No differences in outcomes were reported for a trial comparing internet versus telephone delivery of psycho-education, skills training and symptom assessments [55].

## Discussion

The growing number of publications in this field reflects the increasing demand for strategies that can complement existing services and better support those providing informal care to people living with dementia. A high proportion of the total data based published studies were intervention studies (48%). However, less than half of these identified interventions met initial EPOC design criteria. Most were uncontrolled before-and-after trials, which provide potentially promising feasibility data but require more rigorous testing. Only two studies were rated as methodologically sound on all the EPOC criteria. The evidence base may therefore be compromised by potential bias. Studies were also often characterised by poor response rates and attrition bias [30, 41, 45, 46], and short intervention / follow-up periods [24, 32, 35–37, 39, 46]. Loss to follow up due to death or significant health changes is expected conducting research with older people and people with dementia. However, adequately powered samples that take into account significant attrition rates should be a key consideration for future research.

Telephone counselling delivered over multiple sessions across many months was largely acceptable to caregivers. Mixed findings with regard to caregiver outcomes were reported. In the only telephone intervention study judged as low risk on all EPOC criteria, psycho-education, skills training and health assessments improved depression and behavioural and memory problems [46]. Previous systematic reviews recognise the potential for telephone interventions in supporting people with dementia and their informal caregivers to maintain independence and wellbeing [19]. A lack of time and an inability to leave the person with dementia alone are major barriers to caregivers participating in face-to-face interventions. Telephone counselling may be a more feasible support option, but may still require more resources than are readily available. This may limit sustainability of such interventions.

In the only study of computer-based interventions to meet all EPOC criteria, psychoeducation with strategic access to clinicians via coaching support improved depression and anxiety [28]. Increasingly, innovations in mobile and tablet technology are being used to adapt care for caregivers of people with physical or cognitive impairment. Caregivers can access resources any time of the day, as often as they want. Multiple information formats can be adapted for health literacy or language. Content can also be readily updated or modified to incorporate other critical issues. However, success is reliant on the extent to which the intervention is actually being used. Differences between adopters and non-adopters were reported [25, 33, 52]; suggesting the need for greater consumer input during development. Attitudes towards technology may influence whether caregivers begin to use programs; while perceived competence may influence whether caregivers continue to use it [58].

### Implications for policy and practice and directions for future research

Computer- and telephone-delivered interventions may assist in overcoming some of the deficits in current dementia care. Interventions that include psycho-education via multiple formats, practical strategies and skills training to manage care, and peer and/or clinician contact hold promise. There are a number of potential advantages of these types of interventions. The reach of these interventions is significant and growing each year [59]. They can be delivered any time of the day, and from the comfort of the individual’s home. This has particular advantages for caregivers of people with dementia with severe cognitive deficits and/or limited mobility, or those with poor access to specialised services or professionals [8, 60–62]. Features can be tailored for health literacy and cognitive deficits. For instance, information delivered via computer can be presented in written or audio-visual formats. Algorithms can tailor information according to user needs and preferences. Interventions can augment standard dementia care and improve efficiency of resource utilisation. Providers may supplement face-to-face consultations with interventions, or replace for those caregivers for whom face-to-face interventions are not feasible.

Despite these potential benefits, methodologically rigorous trials are needed to further identify the components of interventions effective in improving outcomes. Given evidence indicating dose effects, strategies to encourage uptake and continued utilisation of the intervention are crucial to ensure sufficiently exposure to achieve improvements. Proactive, personal and detailed invitations from clinicians and reminders have been shown to improve uptake of interventions and adherence [63–65]. It may also be useful to explore which of these strategies are most effective in maximising uptake and engagement. Interventions were rarely tested in other under-served populations, including ethnic minorities [55]. Few examined whether the intervention improved outcomes for the person living with dementia. It may be that providing education and skills training to carers may also result in beneficial effects for the person whom they support. Future research should further explore this possibility.

### Limitations of review

Although the strategy was developed with the assistance of an academic medical librarian, indexing in this rapidly changing field may not have captured all relevant studies and we did not include non-published studies or grey literature. The authors only used the available reporting of research methods from the included publications to determine study quality ratings. Some quality criterion may have been met but received an ‘unclear’ rating when not adequately reported, potentially underestimating study quality. The review was limited to include only those studies that met EPOC criteria which restricted the study sample. While insights on the effectiveness of interventions may be obtained from studies using designs excluded by these criteria, these restrictions increase the likelihood of including studies with high quality evidence.

## Conclusions

Interventions delivered via technology mediums, such as telephone and computer, have the potential to provide information and resources to improve outcomes of caregivers of people with dementia. However, more high-quality intervention trials are required to make clear recommendations about which interventions are most effective in improving caregiver outcomes. Strategies are also needed to maximise utilisation of interventions. Interventions which provide practical strategies to upskill caregivers in managing the care of the person with dementia and their own wellbeing, access to peer and/or clinician support and advice and target the person with dementia and their caregiver as a dyad should be explored.

## Additional files

Additional file 1: Search terms. The search terms used in the search strategy. (DOCX 14 kb)

Additional file 2: Summary table of outcomes. A summary of each of the primary and secondary outcomes assessed in each intervention. (DOCX 96 kb)

## Abbreviations

ADL: Activities of daily living
CBT: Cognitive behaviour therapy
EPOC: Effective practice and organisation of care
NRCT: Non-Randomised controlled trial
RCT: Randomised controlled trial
