# Supplementary material for: Computer and telephone delivered interventions to support caregivers of people with dementia: a systematic review of research output and quality
Source: BMC Geriatr. 2017 Nov 16;17:265. doi: 10.1186/s12877-017-0654-6 (PMC5691399; doi:10.1186/s12877-017-0654-6)
Supplement: Supplementary file 1 — Search terms. The search terms used in the search strategy. (DOCX 14 kb) [file 12877_2017_654_MOESM1_ESM.docx]

Database: MEDLINE 1946 to Present with Daily Update

Search Strategy:

--------------------------------------------------------------------------------

1     exp Dementia/

2     exp Cognition Disorders/

3     (dement* or alzheimer*).tw.

4     1 or 2 or 3

5     Caregivers/

6     (caregiv* or care giv* or carer*).tw.

7     (support person* or wife or wives or husband* or next of kin* or significant other* or couple or dyad* or partner* or spouse*).tw.

8     exp Family/

9     5 or 6 or 7 or 8

10     4 and 9

11     4 or 10

12     Telemedicine/ or Internet/

13     Online systems/

14     User-computer interface/

15     Attitude to computers/

16     Therapy, computer assisted/

17     Remote consultation/

18     (teleconsultation* or "mobile telemedicine").tw.

19     Wireless technology/

20     Cell phones/ or smart phone/

21     Mobile applications/

22     Microcomputers/ or Computers, handheld/

23     (ehealth or mhealth or "web based" or online or internet or "web-guided" or "interactive health communication* application*").tw.

24     12 or 13 or 14 or 15 or 16 or 17 or 18 or 19 or 20 or 21 or 22 or 23

25     11 and 24

26     limit 25 to (english language and yr="1996 -Current")

27     limit 26 to (case reports or clinical conference or comment or editorial or letter or news)

28     26 not 27
