# Supplementary material for: Computer and telephone delivered interventions to support caregivers of people with dementia: a systematic review of research output and quality
Source: BMC Geriatr. 2017 Nov 16;17:265. doi: 10.1186/s12877-017-0654-6 (PMC5691399; doi:10.1186/s12877-017-0654-6)
Supplement: Supplementary file 2 — Summary table of outcomes. A summary of each of the primary and secondary outcomes assessed in each intervention. (DOCX 96 kb) [file 12877_2017_654_MOESM2_ESM.docx]

| **Author, date** | **Depression** | **Emotion/mental health** | **Anxiety** | **Stress, guilt** | **Self- efficacy** | **Burden** | **Satisfied** | **Physical function/ADLs** | **Knowledge** | **Quality of life** | **Social support** | **General health** | **Memory, behaviour problems** | **Goal setting** | **Positive aspects /needs** | **Health care costs** |
| --- | --- | --- | --- | --- | --- | --- | --- | --- | --- | --- | --- | --- | --- | --- | --- | --- |
| **Telephone** |  |  |  |  |  |  |  |  |  |  |  |  |  |  |  |  |
| Au, 2015^1^ | + | + |  |  |  |  |  |  |  |  |  |  |  |  |  |  |
| Au 2014^2^ | + |  |  |  | ND |  |  |  |  |  |  |  |  |  |  |  |
| Chang 1999^3^ | + | ND | ND |  |  | ND |  | ND |  |  |  |  |  |  |  |  |
| Connell 2009^4^ |  |  |  | + |  |  |  | + |  |  |  |  |  |  |  |  |
| Davis 2011^5^ | ND |  |  | + |  | ND | + | ND |  |  |  |  |  |  |  |  |
| Glueckauf 2007^6^ | ND |  |  |  | ND | ND | + |  |  |  |  |  |  |  |  |  |
| Martindale 2013^7^ | ND |  |  |  |  | ND |  |  |  |  | ND | ND | ND |  |  |  |
| Tremont 2008^8^ | ND |  |  |  |  | + |  |  | ND |  | ND | ND | + |  |  |  |
| Tremont 2015^9^ | + |  |  |  | ND | ND |  |  |  | ND |  |  | + |  | + |  |
| Van Mierlo 2012^10^ |  | + |  |  |  | + |  |  |  |  |  |  |  |  |  |  |
| Wilz 2011^11^ |  |  |  |  |  |  |  |  |  |  |  |  |  | + |  |  |
| Wilz 2016^12^ | + | + |  |  |  |  |  | + |  |  |  | + |  |  |  |  |
| Goodman 1990^13^ |  | ND |  |  |  | ND | ND |  | + |  | + |  | ND |  |  |  |
| Winter 2006^14^ | ND |  |  |  |  | ND |  |  |  |  |  |  |  |  | ND |  |
| Wray 2010^15^ |  |  |  |  |  |  |  |  |  |  |  |  |  |  |  | + |
| **Computer** | | | | | | | | | | | | | | | | |
| Beauchamp 2005^16^ | + |  | + | + | + | + |  |  |  |  |  |  |  |  | + |  |
| Blom 2015^17^ | + |  | + |  |  |  |  |  |  |  |  |  |  |  |  |  |
| Brennan 1995^18^ | ND |  |  |  |  | ND |  |  |  |  | ND |  |  | DM |  |  |
| Cristancho 2015^19^ | ND |  |  | ND | ND | ND |  |  | + |  |  | ND | ND |  |  |  |
| Lai 2013^20^ | ND |  | ND |  |  | ND |  |  | + | ND |  |  |  |  |  |  |
| Nunez 2016^21^ | + |  |  |  |  |  |  |  |  |  |  |  |  |  |  |  |
| Pagán-Ortiz 2014^22^ |  | ND |  |  |  | ND |  |  |  |  | ND |  |  |  | + |  |
| Torkamani 2014^23^ | ND | ND |  |  |  | ND |  |  |  | + |  |  |  |  |  |  |
| Van der Roest 2009^24^ |  |  |  |  |  |  |  |  | ND | ND |  |  |  |  | + |  |
| Van Mierlo 2015^25^ |  |  |  | ND |  |  |  |  |  | ND |  |  |  |  | - |  |
| [Czaja](http://www.sciencedirect.com/science/article/pii/S1064748113001668) 2013^26^ | ND |  |  |  |  | + |  |  |  |  | + |  |  |  | + |  |
| Eisdorfer 2003^27^ | + |  |  |  |  | ND |  |  |  |  | ND |  |  |  |  |  |
| Finkel 2007^28^ | + |  |  |  |  | + |  |  |  |  | ND | ND |  |  | + |  |
| Hicken 2016^29^ | - | + |  |  | - |  |  |  |  |  |  |  |  |  |  |  |
| Mahoney 2001 and 2003^30,31^ | + |  | + |  |  |  |  |  |  |  |  |  | + |  |  |  |
| Marziali 2006^32^ | ND | ND |  |  |  |  |  |  |  |  | ND | ND |  |  |  |  |
| Marziali 2011^33^ | ND | + |  |  | ND |  |  |  |  |  |  |  |  |  |  |  |
| Steffen 2016^34^ | + |  |  |  | + |  |  |  |  |  |  |  |  |  |  |  |

+ = significant effect for the intervention group; ND=no difference between groups; - = significant effect for the control group
